# LncRNA SNHG14 promotes hepatocellular carcinoma progression via H3K27 acetylation activated PABPC1 by PTEN signaling

**DOI:** 10.1038/s41419-020-02808-z

**Published:** 2020-08-03

**Authors:** Hui Zhang, Hong-Bo Xu, Erxat Kurban, Hong-Wu Luo

**Affiliations:** Department of Hepatobiliary Surgery, Third Xiangya Hospital, Central South University, Changsha, 410013 Hunan PR China

**Keywords:** Cancer models, Cancer

## Abstract

Hepatocellular carcinoma (HCC) is the fifth most common cancer worldwide. LncRNA small nucleolar RNA host gene 14 (SNHG14) functions as an oncogene in a variety of cancers. However, the role of SNHG14 in HCC remains elusive. The aim of this study is to unravel the functional role and regulatory mechanism of SNHG14 in HCC. A cohort of 40 HCC tumor tissues and paired adjacent normal tissues were collected. Histopathological changes were analyzed by hematoxylin and eosin and immunohistochemistry. qRT-PCR and western blotting were performed to determine the levels of SNHG14, PABPC1, and PTEN signaling molecules. CCK-8, immunofluorescence, and colony formation assays were conducted to monitor cell proliferation. Wound healing and tube formation assays were employed to determine cell migration and angiogenesis. ChIP assay was performed to investigate the enrichment of H3K27 acetylation in PABPC1 promoter. Xenograft mice model was constructed to further verify the SNHG14/PABPC1 axis in vivo. SNHG14 was highly expressed in HCC tissues and cells, which promoted cell proliferation, migration, and angiogenesis in Hep3B and HepG2 cells. PABPC1 functioned as a downstream effector of SNHG14. SNHG14 dramatically induced upregulation of PABPC1 via H3K27 acetylation. In addition, SNHG14/PABPC1 promoted cell proliferation and angiogenesis via PTEN signaling pathway in vitro and in vivo. SNHG14 promoted cell proliferation and angiogenesis via upregulating PABPC1 through H3K27 acetylation and modulating PTEN signaling in the tumorigenesis of HCC.

## Introduction

Hepatocellular carcinoma (HCC), the primary malignancy of liver, is the fifth most common cancer and the third deadliest cancer worldwide^1^. Currently, liver transplantation and surgical resection remain the principal treatment of HCC. For the treatment of advanced HCC, sorafenib is the most commonly used systemic medication^2,3^. Despite the recent advances in liver transplantation and surgery, the prognosis of HCC remains poor. Patients with HCC who are diagnosed in late stages might be excluded from receiving a liver transplant or surgical resection. In addition, high recurrence and metastasis are also associated with poor prognosis^2^. Therefore, it is critical to unravel the underlying mechanism and identify the novel therapeutic targets for HCC.

In recent years, emerging evidence indicates that long noncoding RNAs (lncRNAs, transcripts > 200 nucleotides in length) play important roles in a variety of biological processes at epigenetic, transcriptional, and posttranscriptional levels^4^. More important, the critical roles of lncRNAs in tumorigenesis and cancer progression gained increasing attentions^5,6^. Previous studies have demonstrated that small nucleolar RNA host gene 14 (SNHG14), also known as UBE3A-ATS, exerted oncogenic functions in various types of cancers, including clear cell renal cell carcinoma, gastric cancer, non-small cell lung cancer (NSCLC), cervical cancer, breast cancer, and bladder cancer^7–12^. For instance, SNHG14 promotes cell proliferation via sponging miR-340 in NSCLC cells^12^. A recent study illustrated that SNHG14 promotes proliferation, invasion, and trastuzumab resistance via modulating poly(A) binding protein cytoplasmic 1 (PABPC1) expression through H3K27 acetylation in breast cancer cells^8^. However, the expression pattern, biological function, and underlying regulatory mechanism of SNHG14 in HCC remain largely unclear.

In the present study, we demonstrated that SNHG14 was significantly upregulated in HCC tissues and cells, which promoted cell proliferation, migration, and angiogenesis via regulating PABPC1 in Hep3B and HepG2 cells. Besides, SNHG14 positively regulated PABPC1 via H3K27 acetylation in the promoter of PABPC1. Functional experiments revealed that SNHG14/PABPC1 axis exerted oncogenic functions via inhibiting PTEN pathway in vitro and in vivo.

## Materials and methods

### Collection of clinical specimens

A cohort of 40 HCC tissues and paired adjacent normal tissues were obtained from patients with HCC in The Third Xiangya Hospital of Central South University. The study was approved by the Ethics Committee of The Third Xiangya Hospital of Central South University and consents from all patients were obtained. The relationship between SNHG14 expression and clinical characteristics of HCC patients is shown in Table 1. All experiments were conducted in accordance with provisions of the Declaration of Helsinki and Good Clinical Practice guidelines. None of the patients received preoperative treatment.

**Table 1:** Relationship between SNHG14 expression and clinical characteristics of HCC patients.

Clinical	No. of cases	SNHG14 expression	
		Low (*n* = 18)	High (*n* = 22)
Age (years)			
<59	15	11	4
≥59	25	7	18
Gender			
Male	21	13	8
Female	19	5	14
Tumor size			
<5 cm	12	10	2
≥5 cm	28	8	20
HBV infection			
Positive	30	11	19
Negative	10	7	3
AFP (μg/L)			
<400	13	3	10
≥400	27	15	12
Histologic grade			
Well and moderate	25	14	11
Low	15	4	11
TNM stage			
I/II	27	12	15
III/IV	13	6	7
PVTT			
Yes	11	2	9
No	29	16	13

### Hematoxylin and eosin (H&E) staining and immunohistochemistry (IHC)

Tumors were harvested and fixed with 4% formaldehyde at 4 °C for 24 h. Tissues were then embedded using paraffin and sectioned serially. The slides were dewaxed and stained with H&E for histopathological analysis. For IHC analysis, paraffin sections were deparaffinized, rehydrated, and subjected to antigen retrieval. Slides were then blocked with 10% normal goat serum and incubated with anti-Ki67 (1:500, Abcam, Cambridge, MA, USA), anti-CD31 antibody (1:100, Thermo Fisher Scientific), PTEN (1:100, Cell Signaling Technology, Beverly, MA, USA), or VEGF (1:100, Cell Signaling Technology) at 4 °C overnight, followed by an incubation with biotinylated secondary antibody and streptavidin-HRP. Immunoreactivity was visualized using AEC solution (Thermo Fisher Scientific).

### Cell culture and lentiviral transfection

Human hepatic cell line L02 cells, human HCC cell lines Hep3B, SMMC7721, Huh7, HepG2, and MHCC-97H cells were the Xiangya Experiment Center (Changsha, China), and had been authenticated before use. Human umbilical vein endothelial cells (HUVECs) were purchased from ATCC (Manassas, VA, USA). Cells were tested without contamination with mycoplasma. Cells were cultured in DMEM containing 10% FBS (Gibco, Thermo Fisher Scientific, Waltham, MA, USA), 100 U/ml penicillin, and 100 μg/ml streptomycin, respectively. Cells were grown in a humidified incubator at 37 °C and 5% CO_2_. A specific PTEN inhibitor, SF1670 (5 μM, 24 h) was purchased from MedChemExpress (Monmouth Junction, NJ, USA).

The full-length of SNHG14 and PABPC1 mRNA were amplified and cloned into the lentivirus vector (named as Lv-SNHG14 and Lv-PABPC1) for producing the retrovirus in HepG2 cells by GeneChem (Shanghai, China). The negative control vectors were also synthesized (named as Lv-NC). Similarly, the lentivirus containing the short hairpin RNA (shRNA) sequences against SNHG14 and PABPC1 or the short hairpin negative control (shNC) was also obtained from GeneChem. All the vectors contain green fluorescence protein (GFP). Transfection was conducted using Lipofectamine 3000 transfection reagent (Invitrogen, Thermo Fisher Scientific) according to the manufacturer’s instructions. Twenty-four hours after transfection, cells were subjected to evaluate transfection efficiency. Forty-eight hours after transfection, cells were harvested for the following experiments.

### RNA isolation and quantitative PCR (qRT-PCR)

Total RNA were isolated from tissues and cells using TRIzol reagent (Invitrogen). First-strand cDNA was synthesized using Reverse Transcriptase SuperScript III (Invitrogen). The following primers were used in this study: SNHG14 (forward: 5′-GGGTGTTTACGTAGACCAGAACC-3′, reverse: 5′-CTTCCAAAAGCCTTCTGCCTTAG-3′); PABPC1 (forward: 5′-AGCAAATGTTGGGTGAACGG-3′, reverse: 5′-ACCGGTGGCACTGTTAACTG-3′); and GAPDH (forward: 5′-GAAGGTGAAGGTCGGAGTC-3′, reverse: 5′-GAAGATGGTGATGGGATTTC-3′). qRT-PCR was conducted using Power SYBR Green Master Mix (Applied Biosystems, Thermo Fisher Scientific) and ABI PRISM 7500 real-time PCR system (Applied Biosystems) according to the manufacturer’s instructions. GAPDH was used as an internal control. The specificity of the fluorescent signal was verified by both melting curve and gel electrophoresis. The expression level of the target gene was determined using 2^−ΔΔCT^ method.

### Cell proliferation assay

Cell proliferation was determined using Cell Counting Kit-8 (CCK-8) (Beyotime, Haimen, China) according to the manufacturer’s instructions. In brief, cells (2 × 10^3^ cells per well) were seeded in 96-well plates 24 h prior to the lentiviral transfection. At 48 h after transfection, 10 μl CCK-8 solution was added into each well and incubated for 1 h at 37 °C. Absorbance was measured at a wavelength of 450 nm by the use of microplate reader (Molecular Device, Menlo Park, CA, USA). All experiments were performed in triplicate.

### Immunofluorescence microscopy

Hep3B and HepG2 cells were fixed in 4% paraformaldehyde and permeabilized with 0.1% Triton X-100 for 10 min. Cells were then rinsed with PBS and blocked with 10% normal goat serum for 1 h. This is followed by an incubation with anti-Ki67 antibody (1:500, Abcam, Cambridge, MA, USA) at 4 °C overnight. Cells were rinsed with PBS before incubation with Alexa Fluor 594 conjugated secondary antibody (Molecular Probes, Thermo Fisher Scientific). Slides were mounted in Prolong Gold Antifade reagent with DAPI (Thermo Fisher Scientific). Images were obtained using Nikon Fluorescence Microscope (Nikon Instruments, Inc.).

### Colony formation assay

Hep3B and HepG2 cells were seeded on six-well plates (0.5 × 10^3^ cells per plate) and subjected to lentiviral transduction. Cells were then cultured for 14 days. Medium was replaced every 3 days. The colonies were fixed with 10% formaldehyde and stained with 0.1% crystal violet. Viable containing at least 50 cells were counted. All experiments were repeated at least three times.

### Wound healing assay

Cell migration was evaluated by performing wound healing assay. Hep3B or HepG2 cells were seeded in six-well plates and grown to 90% confluence. The cell monolayer was lightly scratched using a sterile 200 μl micropipette tip. The floating cell debris was carefully removed. Wound closure was observed and evaluated in five random fields using and inverted microscope (Carl Zeiss, Germany) at 24 h. Triplicate wells for each treatment were examined.

### Tube formation assay

Tube formation assay was performed as previously described^13^. In brief, the conditioned medium was the suspension from Hep3B or HepG2 cells transfected with different lentiviral construct(s) for 48 h. Then, HUVECs (6 × 10^4^ cells per well) were collected and cultured in 24-well plates with the conditioned medium and each well was coated with 50 μl growth-factor-reduced Matrigel (BD Biosciences, San Jose, CA, USA). The capillary-like structures were acquired using Zeiss inverted microscope (Carl Zeiss) 24 h later.

### Western blotting

Protein lysates from tissues or cells were prepared in IP lysis buffer (Pierce, Thermo Fisher Scientific). Protein concentration was determined by BCA Protein Assay (Pierce). Protein extracts were then separated on SDS-PAGE, transferred onto PVDF membrane (Millipore, Billerica, MA, USA) and blocked with 5% nonfat milk. Membrane was then incubated with primary antibodies, including PABPC1 (1:1000, Abcam), PTEN (1:1000, Cell Signaling Technology), VEGF (1:1000, Cell Signaling Technology), PI3K (Cell Signaling Technology), p-PI3K (1:1000, Cell signaling technologies), Akt (1:1000, Cell Signaling Technology), p-Akt (1:1000, Cell Signaling Technology), and β-actin (1:1000, Abcam) at 4 °C overnight. The blots were then incubated with corresponding secondary antibody (Thermo Fisher Scientific) for 1 h and visualized using SuperSignal West Pico PLUS Chemiluminescent Substrate (Pierce).

### Enzyme linked immune-sorbent assay (ELISA)

To examine the alteration of VEGF release in HCC cells from culture medium supernatant, ELISA kit (Thermo Fisher Scientific) was performed according to the manufacturer’s instructions.

### RNA immunoprecipitation (RIP) assay

RIP was performed using Magna RNA-binding protein immunoprecipitation kit (Millipore). Hep3B or HepG2 cells were lysed in complete RNA lysis buffer and incubated in RIP immunoprecipitation buffer containing magnetic beads conjugated with anti-AGO2 antibody or mouse IgG control. The immunoprecipitated RNAs were then isolated by Proteinase K and subjected to qRT-PCR.

### Chromatin immunoprecipitation (ChIP) assay

ChIP assay was performed using Pierce Agarose ChIP kit (Pierce) according to the manufacturer’s instructions. Briefly, Hep3B or HepG2 cells were transfected with LV-SNHG14 or sh-SNHG14, respectively. Cells were cross-linked in 1% formaldehyde and lysed at 48 h post transfection. Chromatin was digested using micronuclease. Sheared DNA was incubated with anti-H3K27ac antibody (Abcam). Normal IgG was used as a negative control. DNA was purified and analyzed by PCR.

### Luciferase reporter assay

PABPC1 promoter region was cloned into pGL-3 luciferase reporter vector (Promega, Madison, WI, USA). Hep3B or HepG2 cells were transiently cotransfected with the aforementioned plasmids and SNHG14 overexpressing vector or shRNA and their negative controls, respectively. Luciferase reporter assay was performed using Dual Luciferase Reporter Assay System (Promega) according to the manufacturer’s instructions. Renilla luciferase activity was used to normalize the differences in transfection efficiency.

### Xenograft study

All animal experiments were undertaken in accordance with the National Institute of Health guidelines for the care and use of laboratory animals, with the approval of the Ethics Committee of The Third Xiangya Hospital of Central South University, Changsha. Eighteen female BALB/c nude mice (6–8 weeks old) were purchased from Slac-Jingda Laboratory Animal (Hunan, China). All mice were housed in a 12 h light/12 h dark cycle and controlled environment, for at least 7 days before the experiments. A total of 1 × 10^7^ HepG2 cells transfected with shNC, sh-SNHG14, or sh-SNHG14 + LV-PABPC1 were suspended in 25% Matrigel in serum-free medium DMEM and injected subcutaneously in the flank of each BALB/c nude mouse, within the principle of random allocation. The investigator was blinded to the group allocation during the experiment. The tumor sizes were measured or subjected to IHC analysis 4 weeks after subcutaneous inoculation.

### Statistical analysis

Data are presented as the means ± standard deviation (SD) of triplicate assays in three independent experiments. All data was in a normal distribution, and variance was similar between the groups that are being statistically compared. One-way analysis of variance followed by Tukey’s post hoc test was used for multiple comparisons. In selected experiments, a two-tailed Student’s *t* test was used for paired comparisons. Statistical analysis was performed using the SPSS17.0 (SPSS Inc., Chicago, IL, USA). Differences were considered significant at *P* < 0.05.

## Results

### SNHG14 is highly expressed in HCC tissues and cells

**Fig. 1:**
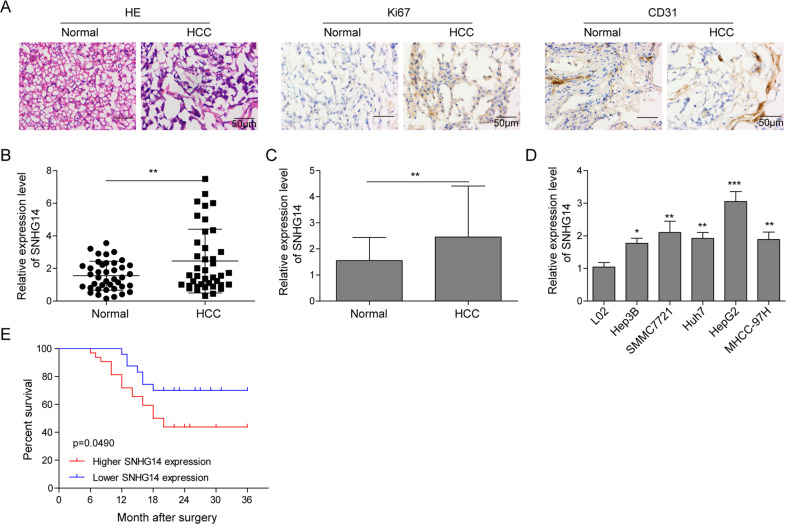
SNHG14 is highly expressed in HCC tissues and cells. **a** H&E staining and IHC analysis of Ki67 and CD31 in HCC and paired adjacent normal tissues. Scale bar, 50 μm. **b**, **c** qRT-PCR analysis was performed to determine the expression of SNHG14 in 40 paired HCC tissues and adjacent nontumor tissues. **d** The expression level of SNHG14 in L02 and different HCC cell lines were determined by qRT-PCR. GAPDH served as an internal control. **e** Kaplan–Meier survival curves for HCC patients with high SNHG14 expression (*n* = 22) and those with low SNHG14 expression (*n* = 18). Data were representative images or were expressed as the mean ± SD of *n* = 3 experiments. *P < 0.05, **P < 0.01, ***P < 0.001.

To explore whether SNHG14 plays a role in the tumorigenesis of HCC, HCC tissues and its paired adjacent normal tissues, we obtained, were firstly employed to evaluate the histopathology. Firstly, we observed that the damaged structure, irregularly arranged cells, stronger expression of Ki67 (proliferation marker) and CD31 (angiogenic marker) in tumor tissues, suggesting that abnormal hyperplasia and angiogenesis in HCC progression (Fig. 1a). Next, we focused on examining the expression pattern of SNHG14. As shown in Fig. 1b, c, a dramatically increased expression of SNHG14 was identified when compared with that of adjacent normal tissues. Then, human normal hepatocytes L02 and five HCC cell lines (Hep3B, SMMC7721, Huh7, HepG2, and MHCC-97H) were employed for further confirmation. The results identified that there was a significantly elevated expression of SNHG14 in five HCC cell lines when compared with L02 (Fig. 1d). Besides, among these five HCC cell lines, relatively low level of SNHG14 was found in Hep3B cells, whereas it was expressed at a relatively high level in HepG2 cells (Fig. 1d). Therefore, these two HCC cell lines were selected for the subsequent gain and loss experiments. Furthermore, SNHG14 in primary tumors form HCC patients (*n* = 40) were determined by qRT-PCR. A cohort of 40 patients with HCC were divided into a SNHG14 high expression group (*n* = 22) and a low expression group (*n* = 18). The results showed that patients with high SNHG14 expression has markedly poorer prognosis with regard to overall survival than those with low SNHG14 expression (Fig. 1e). Taken together, these data suggest that SNHG14 may play an oncogenic role in HCC progression.

### SNHG14 promotes cell proliferation, migration, and angiogenesis in HCC cells

**Fig. 2:**
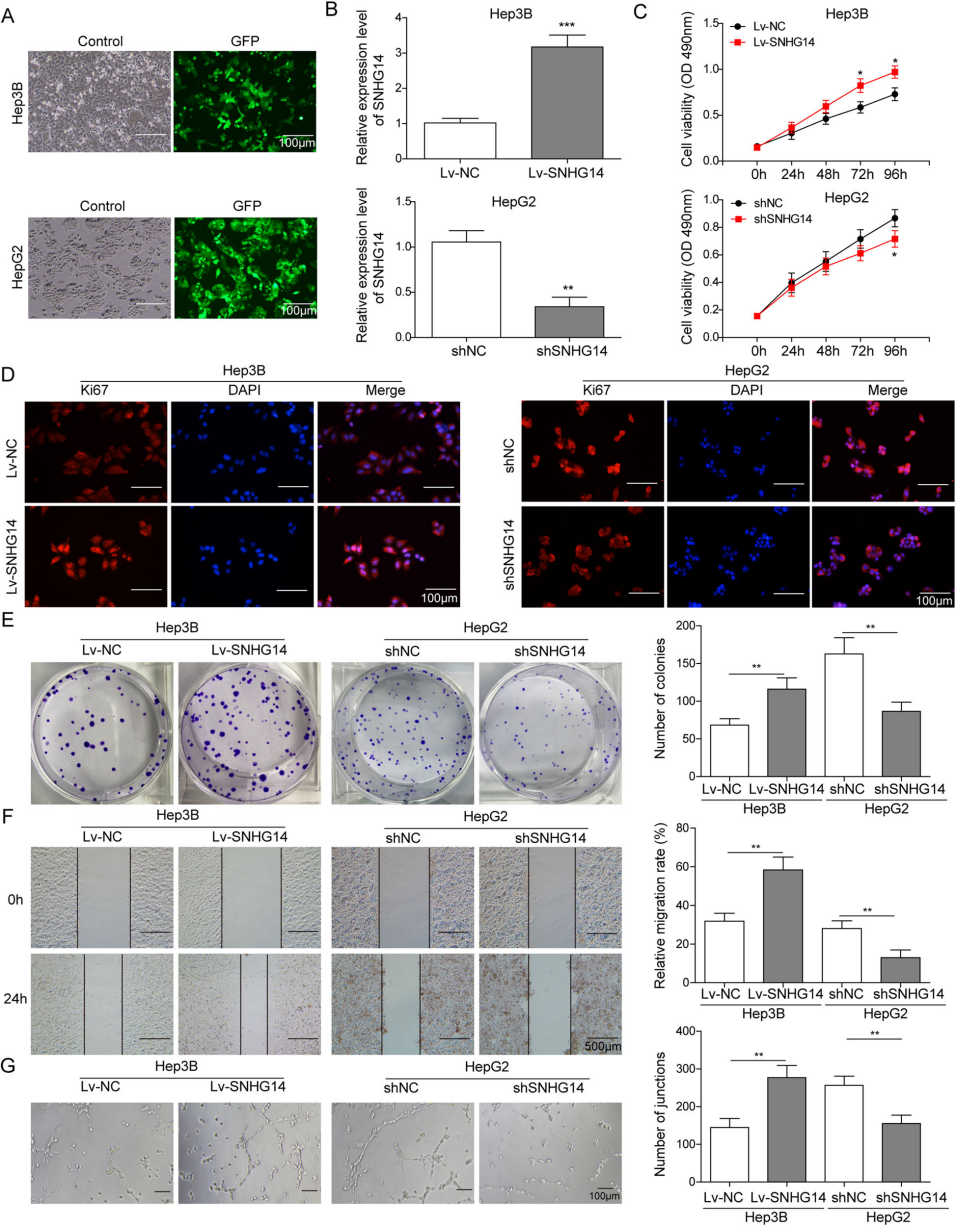
SNHG14 promotes cell proliferation, migration, and angiogenesis in HCC cells. Hep3B and HepG2 cells were transfected with lentiviral vector containing full-length SNHG14 or sh-SNHG14, respectively. **a** The oligonucleotides labeled with GFP green fluorescence was transfected. **b** qRT-PCR assay was subjected to certify the transfection efficiency to examine the change of SNHG14 expression. **c** Cell viability was monitored using CCK-8 assay. **d** Ki67 expression was determined by immunofluorescence. Scale bar, 100 μm. **e** The capacity of cell proliferation was determined by colony formation assay. **f** Cell migration was monitored by wound healing assay. **g** In vitro angiogenesis was quantified by tube formation assays. Data were representative images or were expressed as the mean ± SD of *n* = 3 experiments. *P < 0.05, **P < 0.01, ***P < 0.001.

We further investigated the functional role of SNHG14 in HCC progression in two HCC cell lines. Given that different endogenous SNHG14 expression were found in HCC cells, overexpression and knockdown experiments were performed in Hep3B and HepG2 cells, respectively. The transfection efficiency was measured by visualizing GFP fluorescence. As shown in Fig. 2a, robust GFP fluorescence was detected in the majority of cells. In addition, lentiviral transfection of Lv-SNHG14 successfully increased SNHG14 level in Hep3B cells, whereas sh-SNHG14 dramatically decreased SNHG14 level in HepG2 cells (Fig. 2b). From the data of CCK-8 assays, we observed that overexpression of SNHG14 significantly promoted cell proliferation, while silencing of SNHG14 markedly inhibited cell growth (Fig. 2c). Similarly, immunofluorescent staining of Ki67 also verified the promotion of SNHG14 on cell proliferation (Fig. 2d). Moreover, colony formation assay showed that the number of colonies was notably increased in SNHG14 overexpressing Hep3B, whereas knockdown of SNHG14 caused a suppressed colony formation capacity (Fig. 2e). Furthermore, wound healing assay indicated that SNHG14 overexpression potentiated the capacity of cell migration in Hep3B cells, while knockdown of SNHG14 exerted the opposite effects in HepG2 cells (Fig. 2f). Furthermore, tube fo**r**mation assays were conducted to quantify in vitro angiogenesis. As presented in Fig. 2g, overexpression of SNHG14 induced more branched capillary structures of HUVEC cells. In contrast, broken and shorter tubes were observed within SNHG14 knockdown. Collectively, these findings suggest that SNHG14 may play an oncogenic role of HCC progression.

### PABPC1 acts as a downstream effector of SNHG14 in HCC cells

**Fig. 3:**
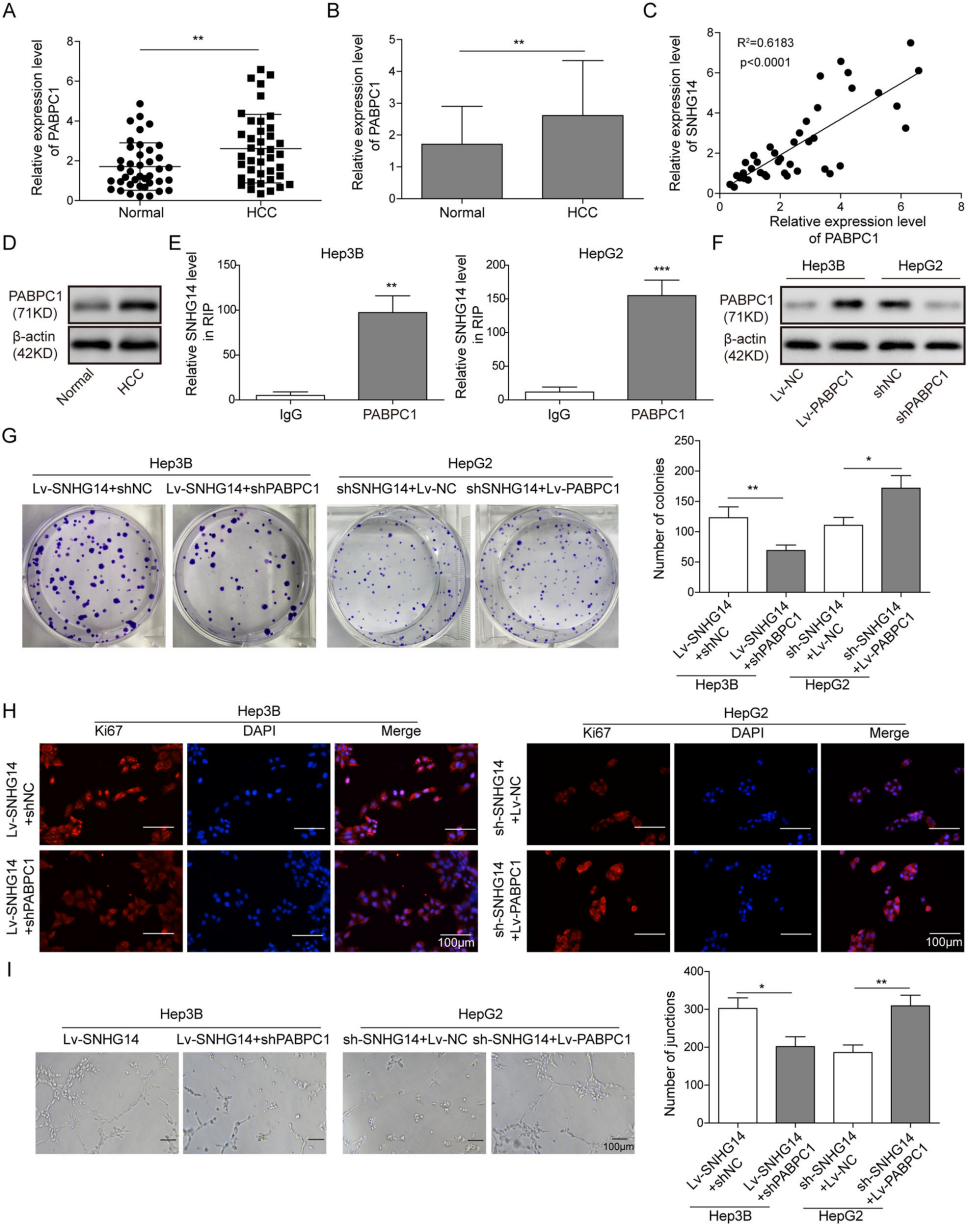
PABPC1 acts as a downstream effector of SNHG14 in HCC cells. **a**, **b** qRT-PCR analysis was presented to detect the expression pattern of PABPC1 in 40 paired HCC tissues and adjacent nontumor tissues. **c** The correlation between SNHG14 and PABPC1 were analyzed by the Pearson correlation coefficient. **d** The protein level of PABPC1 was determined by western blotting. β-actin served as a loading control. **e** The direct interaction between SNHG14 and PABPC1 was verified by RIP. **f** The protein level of PABPC1 was determined by western blotting. **g** Colony formation assay was employed to evaluate the role of PABPC1 for colony formation. **h** Immunofluorescence staining was performed to assess the expression of Ki67 expression in HCC cells. Scale bar, 100 μm. **i** In vitro angiogenesis was quantified by tube formation assays. Data were representative images or were expressed as the mean ± SD of *n* = 3 experiments. *P < 0.05, **P < 0.01, ***P < 0.001.

We further investigated the regulatory mechanism by which SNHG14 exerted its oncogenic role in HCC. Previous study has demonstrated that SNHG14 regulates trastuzumab resistance via targeting PABPC1 expression through H3K27 acetylation (H3K27ac) in breast cancer^8^. Interestingly, in this study, we also identified that PABPC1 was significantly elevated in HCC tissues compared with paired normal tissues (Fig. 3a, b). Subsequently, the Pearson’s correlation coefficient analysis further revealed that SNHG14 was positively correlated with PABPC1 expression in HCC tissues (Fig. 3c). In accordance with the results of qRT-PCR, western blotting also showed that PABPC1 is remarkably upregulated in HCC tissue compared with paired normal tissues (Fig. 3d), indicating that PABPC1 might also serve as a downstream molecule of SNHG14 in HCC. Moreover, the RIP assay further verified the direct interaction between SNHG14 and PABPC1 in which SNHG14 was enriched by PABPC1 in both Hep3B and HepG2 cells (Fig. 3e). In order to investigate whether PABPC1 contributed to SNHG14-mediated oncogenic effects, gain- and loss-of function experiments were conducted. Western blotting confirmed that Lv-PABPC1 and sh-PABPC1 successfully increased and decreased PABPC1 protein levels in Hep3B and HepG2 cells, respectively (Fig. 3f). Immunofluorescent staining and colony formation assays suggested that knockdown of PABPC1 attenuated SNHG14 overexpression-mediated upregulation of Ki67, as well as enhanced clonogenic ability in Hep3B cells. In contrast, overexpression of PABPC1 reversed the suppression of SNHG14 knockdown on cell proliferation in HepG2 cells (Fig. 3g, h). Tube formation assay revealed that sh-PABPC1 abolished the positive effects of SNHG14 overexpression on angiogenesis in Hep3B cells, whereas PABPC1 overexpression exerted a rescue effect on angiogenesis in SNHG14-knockdown HepG2 cells (Fig. 3i). Taken together, these data indicate that PABPC1 acts as a downstream effector of SNHG14 in HCC cells.

### SNHG14 upregulates PABPC1 expression via H3K27 acetylation in HCC cells

**Fig. 4:**
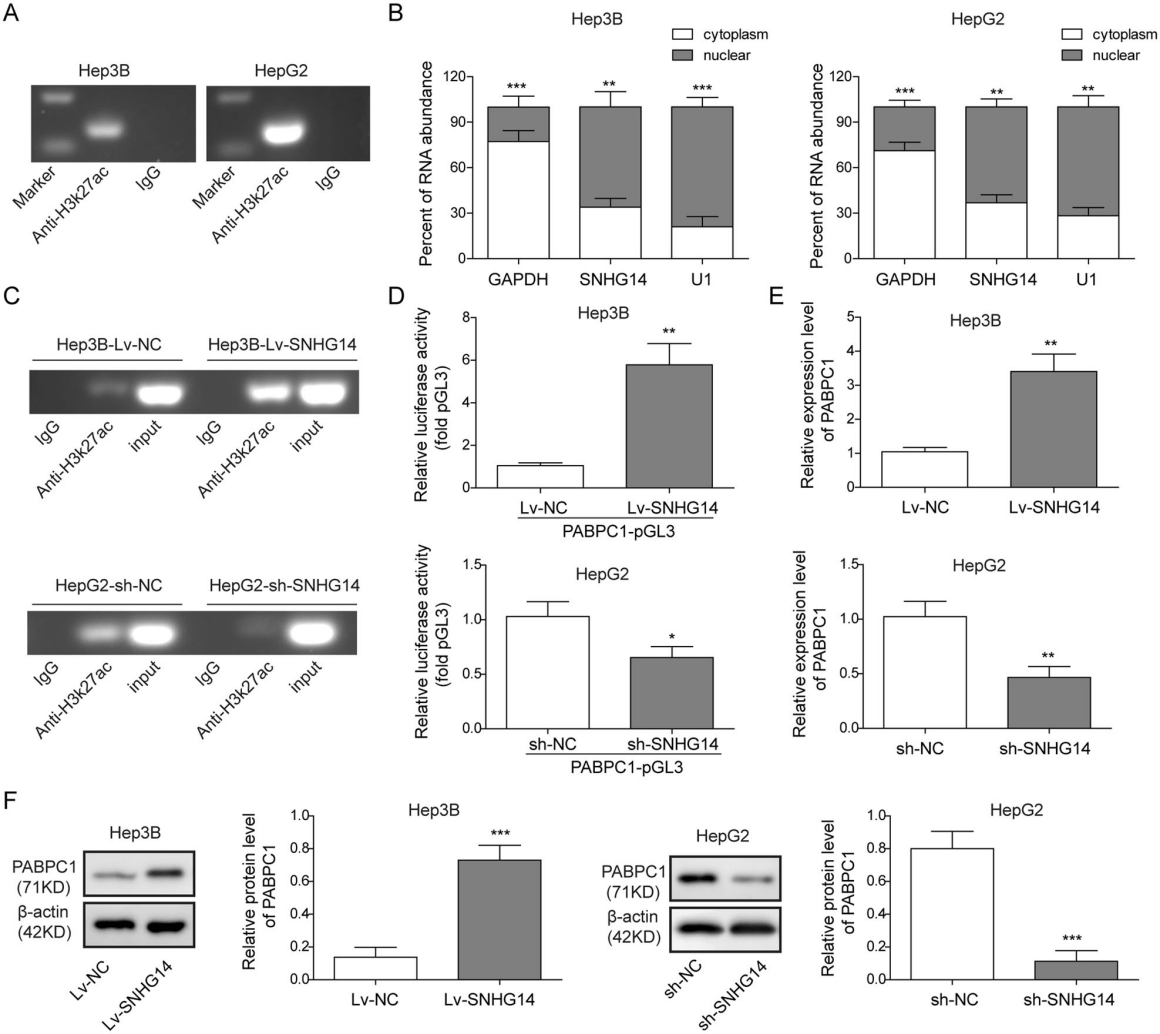
SNHG14 upregulates PABPC1 expression via H3K27 acetylation in HCC cells. **a** The interaction between H3K27ac and PABPC1 promoter region was determined by ChIP assay. **b** The expression of SNHG14 in cytoplasm and nucleus of HCC cells. **c** The effect of SNHG14 on the direct binding between H3K27ac and PABPC1 promoter region was determined by ChIP assay. **d** Relative luciferase activity was determined by dual luciferase reporter assay. Renilla luciferase activity acted as an internal control. **e**, **f** PABPC1 mRNA ad protein levels were determined by qRT-PCR and western blotting. Data were representative images or were expressed as the mean ± SD of *n* = 3 experiments. *P < 0.05, **P < 0.01, ***P < 0.001.

In order to understand the exact mechanism by which SNHG14 modulated PABPC1 expression, we next further examined whether H3K27ac was also involved in regulating PABPC1 transcriptional regulation, according to the previous study. Thus, we performed ChIP assay in Hep3B and HepG2 cells and the results indicated that significant enrichment of H3K27ac was observed in PABPC1 promoter in both HCC cells (Fig. 4a). Predominant nuclear localization of SNHG14 was detected by subcellular fractionation followed by qRT-PCR (Fig. 4b), suggesting that SNHG14 might be involved in H3K27ac-mediated transcriptional regulation of PABPC1. As shown in Fig. 4c, overexpression of SNHG14 promoted the enrichment of H3K27ac in PABPC1 promoter, whereas knockdown of SNHG14 exerted an opposite effect. Moreover, luciferase reporter assay revealed that overexpression of SNHG14 significantly increased the luciferase activity of PABPC1 promoter. Whereas, knockdown of SNHG14 caused the decline of luciferase activity (Fig. 4d). Since it is well established that H3K27ac is antagonistic to gene repression^14^, qRT-PCR was further conducted to test the effect of SNHG14 on PABPC1 expression. Lv-SNHG14 significantly induced PABPC1 mRNA and protein levels in Hep3B cells, while sh-SNHG14 dramatically reduced PABPC1 expression in HepG2 cells (Fig. 4e, f). In short, these data indicate that SNHG14 increased PABPC1 expression via H3K27 acetylation in Hep3B and HepG2 cells.

### SNHG14/PABPC1 promotes cell proliferation and angiogenesis via inhibiting PTEN pathway

**Fig. 5:**
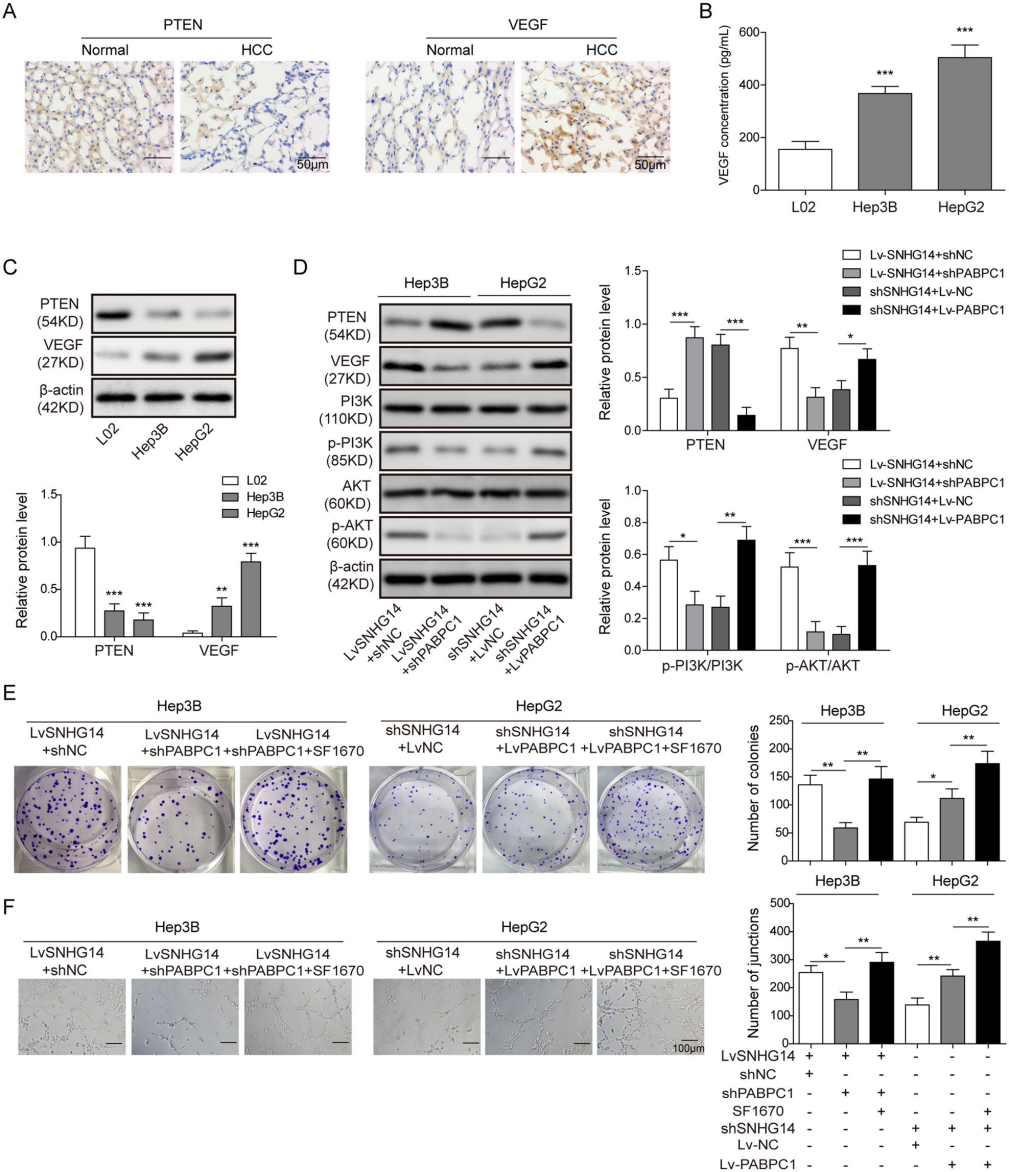
SNHG14/PABPC1 promotes cell proliferation and angiogenesis via inhibiting PTEN pathway. **a** IHC analysis of PTEN and VEGF in HCC and paired adjacent normal tissues. Scale bar, 50 μm. **b** The VEGF level in cell culture supernatant was determined by ELISA assay. **c** Protein levels of PTEN and VEGF in L02 and HCC cells were determined by western blotting. β-actin served as a loading control. **d** Protein levels of the key components of PTEN signaling were determined by western blotting. β-actin served as a loading control. **e** Clonogenic ability was determined by colony formation assay. **f** In vitro angiogenesis was quantified by tube formation assays. Data were representative images or were expressed as the mean ± SD of *n* = 3 experiments. *P < 0.05, **P < 0.01, ***P < 0.001.

Accumulating evidence indicates that PTEN, a well-known tumor suppressor, is frequently lost or mutated in HCC^15–17^. Previous studies have been demonstrated that PTEN is involved in cell proliferation or angiogenesis via negatively regulating PI3K/Akt signaling or VEGF expression, respectively^17,18^. We thus examined whether PTEN pathway was involved in SNHG14/PABPC1-mediated regulation of cell proliferation and angiogenesis in HCC cells. As expected, IHC revealed that PTEN was significantly downregulated in HCC tissues, whereas VEGF was prominently expressed in HCC tissues compared with paired normal tissues (Fig. 5a). ELISA assay further showed that the secreted VEGF was much higher in the cell supernatants of Hep3B and HepG2 cells when compared with that of L02 cells (Fig. 5b). Consistently, downregulated PTEN and upregulated VEGF protein expression were found in Hep3B and HepG2 cells (Fig. 5c). Gain- and loss-of-function experiments also showed that Lv-SNHG14 suppressed PTEN expression, thus leading to upregulation of VEGF and activation of PI3K/Akt signaling in Hep3B cells. In addition, knockdown of PABPC1 inhibited Lv-SNHG4-activated PTEN signaling (Fig. 5d). By contrast, sh-SNHG14 exerted an opposite effect in HepG2 cells, while overexpression of PABPC1 attenuated the effect of sh-SNHG14 (Fig. 5d). Subsequently, functional researches of colony formation and tube formation assays showed that Lv-SNHG14-mediated enhanced clonogenic ability and angiogenesis were partially abolished by sh-PABPC1, while PTEN inhibitor SF1670 further rescued the impaired clonogenic ability and angiogenesis in Hep3B cells (Fig. 5e, f). In contrast to Lv-SNHG14, sh-SNHG14 + Lv-PABPC1 promoted colony formation and angiogenesis when compared with sh-SNHG14 alone, whereas SF1670 abrogated this effect in HepG2 cells (Fig. 5e, f). Taken together, these data suggest that SNHG14/PABPC1 axis may promote cell proliferation and angiogenesis via inhibiting PTEN signaling.

### SNHG14/PABPC1 promotes tumor progression via PTEN signaling in vivo

**Fig. 6:**
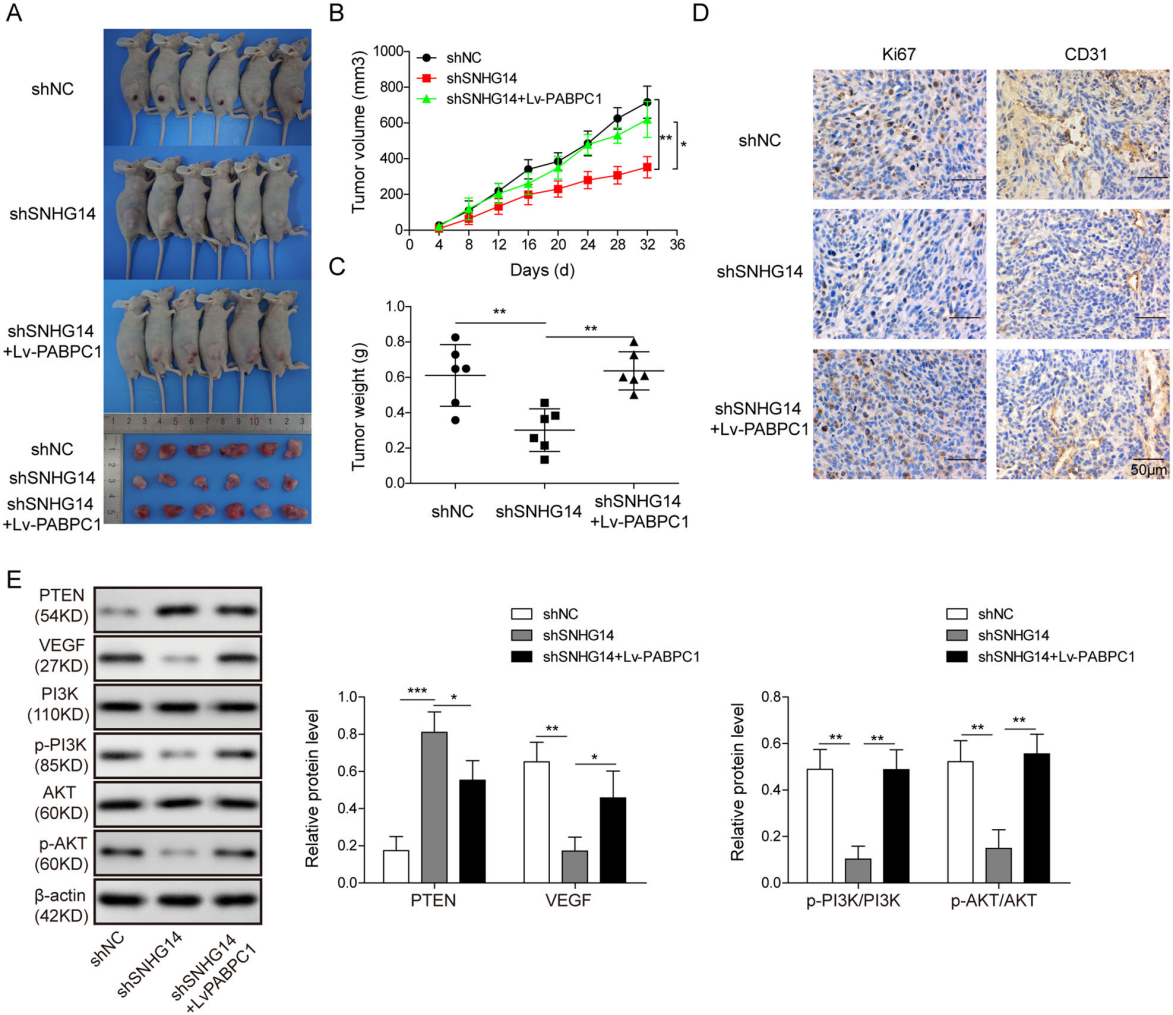
SNHG14/PABPC1 promotes tumor progression via PTEN signaling in vivo. **a** Photographs of tumor that developed in xenograft transplanted nude mouse tumor models in different groups. **b** Quantitative analysis of tumor volumes. **c** Quantitative analysis of tumor weights. **d** IHC analysis of Ki67 and CD31 in tumors of different groups. Scale bar, 50 μm. **e** Protein levels of the key components of PTEN signaling were determined by western blotting. β-actin served as a loading control. Data were representative images or were expressed as the mean ± SD of *n* = 3 experiments. *P < 0.05, **P < 0.01, ***P < 0.001.

To further verify the regulatory network of SNHG14/PABPC1 in vivo, nude mice xenograft models were generated by subcutaneous injection of SNHG14-knockdown HepG2 cells, sh-SNHG14 + Lv-PABPC1-transfected HepG2 cells, or control cells. Tumors were harvested 7 weeks post implantation. We observed that silencing of SNHG14 induced a remarkable reduction in tumor size, while overexpression of PABPC1 impaired the suppression of SNHG14 knockdown on tumor growth (Fig. 6a–c). To delineate the underlying mechanism, the harvested tumors were further subjected to IHC and western blotting analysis. IHC staining further indicated that the dramatically decreased expression of Ki67 and CD31 induced by sh-SNHG14 was reversed by PABPC1 overexpression (Fig. 6d). Consistent with the previous in vitro findings, knockdown of SNHG14 also induced PTEN expression, further leading to reduction of VEGF and inactivation of PI3K/Akt signaling, while the PTEN expression was repressed and the PI3K/Akt signaling was reactivated by overexpression of PABPC1 (Fig. 6e). These in vivo data imply that SNHG14/PABPC1 promotes tumor progression via PTEN signaling in vivo.

## Discussion

In recent years, high-throughput sequencing provides a better understanding of HCC-related lncRNAs. A large number of dysregulated lncRNAs have been identified, and they are associated with a variety of biological processes in HCC, such as cell proliferation, apoptosis, migration, invasion, and angiogenesis^19–21^. Emerging researches have revealed that SNHG family plays important roles in tumorigenesis and the immune escape of cancer via sponging miRNA^22–24^. In HCC, SNHG5 is dramatically upregulated and closely associated with several clinicopathological parameters, such as tumor size, TNM stage, etc. Functional study revealed that SNHG5 exerts its oncogenic effect via miR-26a-5p/GSK3β axis and Wnt/β-catenin signaling in HCC cells^25^. Apart from SNHG5, a more recent study has illustrated the important roles of SNHG3 in epithelial–mesenchymal transition and sorafenib resistance in HCC cells^12^. Several studies have demonstrated that SNHG14 functions as oncogene in various cancers; however, the biological function of SNHG14 in HCC remains largely elusive. In this study, increased expression of SNHG14 was observed in HCC tissues and cell lines. Overexpression and knockdown experiments further revealed the oncogenic role of SNHG14 in cell proliferation, migration, and angiogenesis in Hep3B and HepG2 cells, suggesting that SNHG14 also acts as an oncogene in HCC.

The aberrant expression and critical function of SNHG14 in HCC promotes us to further unravel the underlying regulatory mechanism. Interestingly, our preliminary experiments showed that PABPC1 expression was higher in HCC tissues and cells compared with normal controls. PABPC1, a member of conserved PABPC gene family, plays crucial roles in poly(A) shortening, recruitment of ribosome, and translation initiation via specifically binding to poly(A) tail of mRNA in cytoplasm^26^. More important, previous studies also illustrated that PABPC1 interacts with argonaute 2 (AGO2) and contributes to miRNA-mediated gene silencing in high-grade HCC^27^. In addition to PABPC1-mediated inhibition efficiency of miRNA, our findings indicated that PABPC1 acted as a downstream effector of SNHG14 in HCC cells. Silencing of PABPC1 attenuated SNHG14-induced cell proliferation and angiogenesis in Hep3B cells, whereas overexpression of PABPC1 abrogated the effect of sh-SNHG14 on cell proliferation and angiogenesis in HepG2 cells. Moreover, it is worth noting that AGO2, which is an active part of RNA-induced silencing complex, is essential for miRNA maturation and small RNA-guided gene silencing^28^. In HCC, AGO2 regulates angiogenesis via PTEN/VEGF signaling^29^, strongly suggesting that SNHG14 might modulate angiogenesis via PABPC1/AGO2 in HCC. Further investigation is required to validate this hypothesis.

Histone acetylation is one of the major histone modification type involved in the regulation of chromatin structure remodeling and transcription. It is well established that acetylation neutralizes the positive charge of lysine to unfold the chromatin structure, thus leading to attenuated interaction between DNA and histone, as well as enhanced transcriptional activity^30^. Genome bioinformatics analysis (http://genome.ucsc.edu/) indicated that H3K27ac was highly enriched in the promoter region of PABPC1. ChIP assay confirmed the enrichment of H3K27ac in PABPC1 promoter region in both Hep3B and HepG2 cells. Subsequent subcellular fractionation revealed that SNHG14 was predominantly expressed in nucleus, indicating its potential role in the regulation of gene transcription. Notably, dysregulation of SNHG14 positively regulated PABPC1 expression via H3K27ac in HCC cells. This finding is consistent with a previous study that demonstrated that SNHG14 promotes breast cancer tumorigenesis and chemoresistance via regulating PABPC1 expression through H3K27ac^8^, indicating that SNHG14 might regulate PABPC1 expression by similar mechanism in different types of cancers.

PTEN, a well-known tumor suppressor, is commonly dysregulated in HCC^15,16^. Previous studies have illustrated that PTEN exerted its anti-oncogenic effect via antagonizing PI3K/Akt signaling^18,31,32^. In HCC, the critical contributor of angiogenesis VEGF is frequently upregulated^33^. It has been reported that PTEN regulates angiogenesis and VEGF expression in HepG2 cells^17^. Consistently, our findings showed that PTEN was significantly downregulated, whereas VEGF was dramatically upregulated in HCC tissues compared with normal tissue counterparts. Colony and tube formation assays revealed that PTEN/PI3K/Akt and PTEN/VEGF signaling contributes to SNHG14/PABPC1-regulated cell proliferation and angiogenesis. Activation of PI3K/Akt contributes to cell proliferation, survival, and migration, and VEGF plays an important role in angiogenesis^31,32,34^, indicating that SNHG14/PABPC1 might regulate cell proliferation and angiogenesis via PTEN/PI3K/Akt and PTEN/VEGF signaling, respectively.

In conclusion, SNHG14 promoted cell proliferation and angiogenesis via upregulating PABPC1 through H3K27 acetylation and modulating the PTEN signaling in Hep3B and HepG2 cells.
